# Osteonecrosis of the jaws in patients under osteoporosis treatment: a nine-year experience report

**DOI:** 10.20945/2359-4292-2023-0226

**Published:** 2024-03-28

**Authors:** Efsun Somay

**Affiliations:** 1 Baskent University Faculty of Dentistry Department of Oral and Maxillofacial Surgery Ankara Turkey Department of Oral and Maxillofacial Surgery, Faculty of Dentistry, Baskent University, Ankara, Turkey

**DEAR EDITOR,**

The scholarly work titled "Osteonecrosis of the jaws in patients under osteoporosis treatment: a nine-year experience report" authored by Penoni and cols. (1) was perused with keen interest. The authors are to be commended for their investigation of a seldom-discussed yet consequential complication that adversely impacts patients’ well-being. The authors’ objective was to present a report on the occurrence of medication-induced osteonecrosis of the jaw (MRONJ) in osteoporotic patients over a period of nine years, along with the associated factors that led to osteonecrosis development. The digital records of 6,742 invasive oral procedures performed on patients receiving osteoporosis treatment were analyzed. MRONJ occurred in 0.03% and 0.06% of osteoporosis patients who underwent dental treatment and tooth extractions, respectively. Consequently, the authors concluded that the incidence of MRONJ linked to osteoporosis treatment was extremely low and stated that the established protocols were effective in preventing MRONJ. While the outcomes of this extensive and well-attended investigation hold significant merit within the realm of MRONJ research, I would like to express two areas of concern.

First, the authors reported that the two instances of medication-related MRONJ were associated with tooth extraction and a removable denture, respectively. Notably, there were no reported cases of MRONJ resulting from implant placement, suggesting that dental placement is a safe procedure with respect to MRONJ risk. This discovery resembles the results obtained by Escobedo and cols. (2), who documented that antiresorptive agent usage leads to MRONJ in patients with functional loading implants, and this phenomenon is more prevalent than the incidence rate observed after implant placement surgery. Excessive forces in the prosthetic period, causing bone resorption in the implant area and accompanying periodontal infection and peri-implantitis as one of the precipitating factors for MRONJ (3,4), can explain this phenomenon. Hence, it has been asserted that it may be necessary to prolong the loading time for patients using antiresorptive medications (2). Unfortunately, Penoni and cols. (1) did not provide the window period between implant placement and prosthetic loading. Because prosthetic loading constitutes an integral aspect of implant therapy, it may be worthwhile to conduct a distinct analysis of MRONJ incidence rates after implant placement and the prosthetic phase.

Second, the absence of information regarding the duration of antiresorptive drug usage among patients, despite being acknowledged as a limitation of the study, may compromise the accuracy of assessing the impact of antiresorptive medication use on the incidence of MRONJ. However, this factor must be considered in the evaluation procedure. As reported, the risk of developing MRONJ is higher in patients who have used bisphosphonates for more than four years (5). Thus, the likelihood of MRONJ after dental implant procedures is contingent upon the duration of antiresorptive utilization in addition to individual patient factors. Hence, it could be deemed more suitable to conduct precise analyses based on the antiresorptive category, dosage, and duration of administration to ascertain the exact risk of MRONJ.
